# Increasing postpartum family planning uptake through group antenatal care: a longitudinal prospective cohort design

**DOI:** 10.1186/s12978-018-0644-y

**Published:** 2018-12-17

**Authors:** Jody R. Lori, Meagan Chuey, Michelle L. Munro-Kramer, Henrietta Ofosu-Darkwah, Richard M. K. Adanu

**Affiliations:** 1 Department of Health Behavior and Biological Sciences, University of Michigan, School of Nursing, 400 N. Ingalls Bldg, Ann Arbor, MI 48109 USA; 2 Manhyia District Hospital, Ministry of Health, Kumasi, Ghana; 3 School of Public Health, University of Ghana, PO Box GP4236, Accra, Ghana

**Keywords:** Antenatal care, Family planning, Group antenatal care, Sub-Saharan Africa, Reproductive health

## Abstract

**Background:**

Despite significant improvements, postpartum family planning uptake remains low for women in sub-Saharan Africa. Transmitting family planning education in a comprehensible way during antenatal care (ANC) has the potential for long-term positive impact on contraceptive use. We followed women for one-year postpartum to examine the uptake and continuation of family planning following enrollment in group versus individual ANC.

**Methods:**

A longitudinal, prospective cohort design was used. Two hundred forty women were assigned to group ANC (*n* = 120) or standard, individual care (*n* = 120) at their first ANC visit. Principal outcome measures included intent to use family planning immediately postpartum and use of a modern family planning method at one-year postpartum. Additionally, data were collected on intended and actual length of exclusive breastfeeding at one-year postpartum. Pearson chi-square tests were used to test for statistically significant differences between group and individual ANC groups. Odds ratios and adjusted odds ratios were calculated using logistic regression.

**Results:**

Women who participated in group ANC were more likely to use modern and non-modern contraception than those in individual care (59.1% vs. 19%, *p* < .001). This relationship improved when controlled for intention, age, religion, gravida, and education (AOR = 6.690, 95% CI: 2.724, 16,420). Women who participated in group ANC had higher odds of using a modern family planning method than those in individual care (AOR = 8.063, *p* < .001). Those who participated in group ANC were more likely to exclusively breastfeed for more than 6 months than those in individual care (75.5% vs. 50%, p < .001). This relationship remained statistically significant when adjusted for age, religion, gravida, and education (AOR = 3.796, 95% CI: 1.558, 9.247).

**Conclusions:**

Group ANC has the potential to be an effective model for improving the uptake and continuation of post-partum family planning up to one-year. Antenatal care presents a unique opportunity to influence the adoption of postpartum family planning. This is the first study to examine the impact of group ANC on family planning intent and use in a low-resource setting. Group ANC holds the potential to increase postpartum family planning uptake and long-term continuation.

**Trial registration:**

Not applicable. No health related outcomes reported.

## Plain English summary

Antenatal care (ANC) presents a unique opportunity to influence the adoption of postpartum family planning. Women were assigned into group verses standard, individual care following their first ANC visit with other women of the same gestational age. We examined whether participation in group ANC (intervention group) increased the uptake and continuation of postpartum family planning compared to women receiving standard, individual focused ANC in Ghana. Women who participated in group ANC were more likely to use a modern or non-modern method of contraception one-year postpartum. Our second outcome of interest was length of exclusive breastfeeding. Women participating in group ANC were more likely to report exclusive breastfeeding for longer than six months than women in individual care. This is the first study to examine the impact of group ANC on length of breastfeeding and family planning intent and use in a low-resource setting. Group ANC holds the potential to increase postpartum family planning uptake and long-term continuation.

## Background

A quarter of birth intervals worldwide are less than 24 months with an interval of less than 18 months between pregnancies associated with an increased risk of low birth weight and small size at birth. [1]. A study using pooled data from 52 DHS surveys found infants born within 24 months of an older sibling have a 60% greater chance of dying before their first birthday [2]. Several studies have identified an association between anemia in women and short interpregnancy intervals [3, 4]. A systematic review found strong evidence that folate depletion occurring in the postpartum period contributes to an increased risk of adverse perinatal outcomes for women with short interpregnancy intervals [5].

The global unmet need for contraception in developing countries now stands at 214 million women of reproductive age who wish to avoid a pregnancy but are not using a modern method of family planning [6]. Uptake of postpartum family planning in sub-Saharan Africa remains low [7] while fertility and projected population growth are much higher than in other regions of the world [8]. A study of 57 low- and middle-income countries found modern contraceptive use is the lowest in sub-Saharan Africa (39%) when compared to three other world regions [9]. In Ghana, 30% of currently married women have an unmet need for family planning [10]. A recent study prospectively assessing the unmet need for family planning in the first year following childbirth found an unmet need in 82% of women in Ghana, the highest of the 16 countries included in the study [11].

Researchers have sought to understand what factors influence the adoption of postpartum family planning, specifically how messaging from healthcare providers influence the uptake of family planning methods [7, 12]. Delivering family planning messages in a manner that allows women to process and apply the information, ultimately using it to impact the health of themselves and their families, is of utmost importance. In pregnancy, family planning education is often introduced solely as a component of postpartum education, a time when many additional messages are being given on self-care and newborn care. A recent Cochrane review found evidence on the efficacy of interventions to increase postpartum contraceptive uptake [13] to be of low quality. A renewed understanding of antenatal care (ANC) as central to the reproductive health continuum with the potential for a long-term positive impact on maternal and newborn health has recently been posited [14]. Antenatal care is an essential time to address a woman’s future need for contraception and reproductive health care [15, 16].

Despite the documented benefits, exclusive breastfeeding rates remain low in many low- and middle-income countries [17]. A systematic review found significant increases in exclusive breastfeeding rates resulting from breastfeeding promotion interventions [17]. Breastfeeding education currently takes on many different forms and is received from a multitude of sources. Multiple global initiatives address the importance of exclusive breastfeeding up to 6 months of age, yet exclusive breastfeeding rates have not increased in the past two decades [18, 19].

Antenatal care has been delivered the same way for decades. Transmitting health information in a clinical setting often fails to consider the social and economic circumstances of patients and their health literacy [20], resulting in a failure to achieve the expected impact on health behaviors [21] and outcomes. This divide has contributed to a lack of progress in providing quality care to the most vulnerable populations. If pregnant women do not receive contraceptive health messages in a comprehensible way from their providers, they cannot effectively maximize the benefits. Efforts to enhance the quality and experience of women in ANC through increased interaction with providers has been proposed. This increased interaction aims to engage women as active participants in their health and healthcare.

### Group antenatal care

Group ANC uses a facilitated discussion methodology to deliver care in small groups with 8–12 women of similar gestational age. While group ANC, where all clinical care and educational content is provided in a group setting with trained facilitators, has been delivered and studied in high-resource settings for over a decade, it has only recently been introduced as an alternative to individual care in sub-Saharan Africa. Two randomized controlled trials of group ANC in the U.S. found women assigned to group care had significantly better antenatal knowledge, greater satisfaction with care, and were less likely to have a preterm birth than those in individual, standard care. In addition, these studies demonstrated more favorable birth, neonatal, and reproductive outcomes in the intervention groups [22, 23]. Although the experimental design of the studies from high-resource countries were scientifically rigorous, findings cannot be generalized to low-resource countries with low literacy rates and high rates of maternal and newborn morbidity and mortality. In the U.S., Australia, and the Netherlands, group ANC has improved outcomes in birth weight, preterm birth rates, satisfaction with care, and breastfeeding initiation [22, 24, 25]. In sub-Saharan Africa, data from two small studies found ANC delivered in groups to be acceptable and feasible to both women and providers in Ghana, Tanzania, and Malawi [26, 27]. Previous findings from this study found significant differences immediately postpartum between women enrolled in group verses individual ANC for birth preparedness and complication readiness, knowledge on preventing problems before delivery, understanding when to access care, and greater understanding of the components of breastfeeding and lactational amenorrhea for birth spacing immediately postpartum [28]. A recent systematic evidence synthesis of group ANC in 16 low- and middle-income countries identified consistent attributes across several different models with recommendations for a composite generic model [29]. Additional group ANC studies are currently underway in several low-income countries with a recommendation from WHO (2016) for increased research into the group ANC [30] model.

This study compares women enrolled in group ANC with women receiving standard, individual focused ANC at a busy urban clinic in Ghana, to: (1) Compare women’s intent to use family planning immediately postpartum, and actual use at one-year postpartum, and (2) Compare exclusive breastfeeding for a minimum for six months.

## Methods

### Study design

Our study used an observational, prospective cohort design with intervention and control groups. Women were enrolled in the study at their first ANC visit and followed for one-year postpartum. Details of the study protocol are described elsewhere [26, 28]. Institutional review board approval for the study was obtained from the University of Ghana Noguchi Memorial Institute for Medical Research; the Kwame Nkrumah University of Science and Technology Committee on Human Research, Publications and Ethics; and the University of Michigan’s Institutional Review Board.

### Materials

The materials developed for the group ANC model have been described elsewhere [26, 28]. Briefly, educational content was delivered through picture cards, role-play, story-telling, and teach-back to small groups of women with similar gestational age. Women remained in the same group throughout their pregnancy developing peer support with the women in the group. Women in both group and individual care were encouraged to attend seven ANC visits. Educational content was similar between both groups with one complete ANC visit dedicated to family planning and exclusive breastfeeding as a lactational amenorrhea method for birth spacing.

### Study setting and sample

A facility-driven convenience sample of 240 women were initially enrolled in the study. We conducted the study at a busy urban district hospital in the Ashanti Region of Ghana outside one of the most populous cities. Women enrolling for their first antenatal visit, over the age of 18 years and less than 14 weeks gestation, were approached by a Ghanaian research assistant (RA). The RA described the purpose of the research, the participant’s ability to refuse to participate without any negative consequences or withdraw from the study at any time, the risks and benefits of participation, and the nature of the research. After informed consent was obtained, every other woman was then assigned to group or individual ANC for her next visit.

### Measurements and data collection

The outcomes of interest were use of any family planning at 1 year postpartum, use of a modern method of family planning at one year postpartum and exclusive breastfeeding at 6 months postpartum. Demographic data were collected on all women at the time of enrollment in the study. Data on intended breastfeeding length and intent to use family planning were collected immediately postpartum through face-to-face surveys or via cellphone by a Ghanaian RA using a structured survey. Longitudinal data on postpartum family planning use and length of exclusive breastfeeding for six months were collected one-year postpartum using a 9-question survey. Methods considered to be modern were injectable contraceptives, an intrauterine device, implant, or condoms. Non-modern methods included the lactational amenorrhea method, abstinence, or withdrawal. The surveys at one-year postpartum were conducted via cellphone by a Ghanaian research assistant. Participants were given a canvas carrying bag as a token of appreciation.

### Data analysis

Demographic information of participants was analyzed with distribution frequencies. Pearson chi-square tests were used to test for statistically significant differences in sociodemographic characteristics between women in group and individual ANC groups who provided follow-up data, as well as differences in characteristics between women who did and did not provide follow-up data. Rates of overall contraceptive use and breastfeeding were analyzed with distribution frequencies. Odds ratios and adjusted odds ratios for contraceptive use and breastfeeding were calculated by ANC type using logistic regression. Models were controlled for age of participant, gravida, religion, and highest level of education. Regression models were developed using complete case analysis. Analyses were conducted in SPSS v.24. All *p*-values were set at .05.

## Results

### Demographic information

**Fig. 1:**
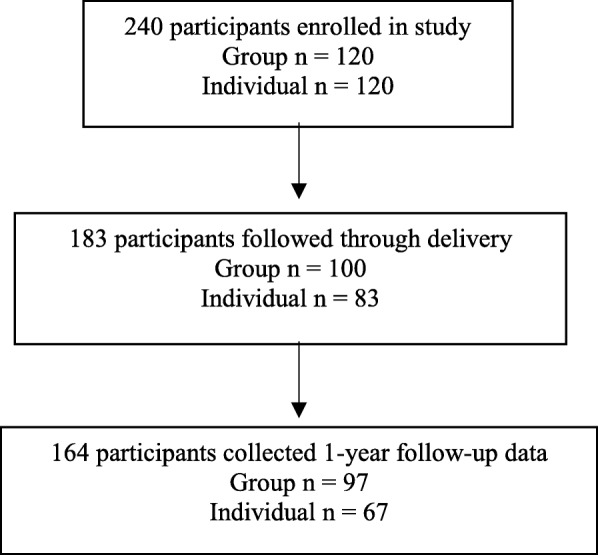
Flow diagram

We were able to locate 164 women (68.3%) at one-year postpartum (Fig. 1).The majority of participants were Christian, multigravida, had completed at least junior high school, and were between the ages of 17–35 years old (M = 27.5 years old). Attrition analyses indicate women who were lost to follow-up were not statistically different than women who provided follow-up data other than they were more likely to have participated in individual prenatal care (<.001). In women who provided follow-up data there were no statistical differences in demographics between those who participated in group and those who participated in individual prenatal care (Table 1).

**Table 1:** Sociodemographic Information

	Total N (%)	Individual antenatal care	Group antenatal care	*p*-value comparing the two groups	*p*-value comparing those with follow-up data and those lost to attrition
All Participants	164 (100%)	67 (40.9%)	97 (59.1%)	–	<.001
Age				.463	.508
17-25yo = 0	69 (42.1%)	32 (47.8%)	37 (38.1%)		
26-35yo = 1	76 (46.4%)	29 (43.3%)	47 (48.5%)		
Over 35 = 2	19 (11.5%)	6 (8.9%)	13 (13.4)		
Gravida				.724	.140
Primip	29 (17.7%)	11 (16.4%)	18 (18.6%)		
Multip	135 (82.3%)	56 (83.6%)	79 (81.4%)		
Religion				.133	.210
Christian	100 (62.9%)	37 (56.1%)	63 (67.7%)		
Muslim	59 (37.1%)	29 (43.9%)	30 (32.3%)		
Education				.373	.304
Primary	29 (20.7%)	20 (23.8%)	9 (16.1%)		
Jr. High School	63 (45.0%)	34 (40.5%)	29 (51.8%)		
Sr. High School	26 (18.6%)	18 (21.4%)	8 (14.3%)		
Tertiary Schooling	22 (15.7%)	12 (14.3)	10 (17.8%)		

### Use of family planning

Overall, 57.6% of women were using no method of contraception at one-year postpartum, 35.8% were using a modern method, and 6.6% were using a non-modern method. Women who participated in group ANC were more likely to demonstrate intention to use family planning immediately postpartum than those who were in individual care (63.0% vs. 31.6%, X^2^ = 16.49, *p* < .001), and one year post-delivery were more likely to actually be using a family planning method (59.1% vs 19%, X^2^ = 26.309, *p* < .001) (Table 2).

**Table 2:** Family planning intent, use at one-year, and exclusive breastfeeding at 6 months postpartum by type of ANC attendance

Intent to Use Family Planning Immediately Postpartum (*n* = 168)*	Type of Antenatal Care		
	Individual n (%)	Group n (%)	*p*-value
No	52 (68.4)	34 (37.0%)	< 0.001
Yes	24 (31.6%)	58 (63.0%)	
Using Family Planning One-Year Postpartum (*n* = 151)*	Individual	Group	*p*-value
No	51 (81.0%)	36 (40.9%)	< 0.001
Yes	12 (19%)	52 (59.1%)	
Modern	8 (66.7%)	46 (88.5%)	
Non-Modern	4 (33.3%)	6 (11.5%)	
Exclusive Breastfeeding Longer than 6 Months (*n* = 158)*	Individual	Group	*p*-value
No	32 (50%)	23 (24.5%)	0.001
Yes	32 (50%)	71 (75.5%)	

*Missing data and “other” responses excluded from analysis

Women in both individual and group ANC who reported they intended to use family planning immediately postpartum were more likely to be using a family planning method at one-year postpartum than those with no immediate postpartum intent (52.1% vs. 33.3%; X^2^ = 5.191, *p* = .023) (Table 3).

**Table 3:** Intent to use vs. using family planning at one-year postpartum

Using Family Planning One-Year Postpartum (*n* = 145)	Reported Immediate Postpartum Intention to Use Family Planning		
	Yes	No	*p*-value
No	35 (47.9%)	48 (66.7%)	.023
Yes	38 (52.1%)	24 (33.3%)	

Women who participated in group ANC had higher odds of using a modern or non-modern method of contraception at one-year postpartum, controlling for intention, age, gravida, religion, and education (AOR = 6.690, 95% CI: 2.724, 16.420).

Women who stated immediately postpartum that they intended to use contraception had higher odds of using a family planning method (modern or non-modern) one-year postpartum than those with no stated intention (OR = 2.171, 95% CI: 1.109, 4.250). There was no significant difference in family planning use based on age of participant, gravida, religion, or highest level of education. However, when controlling for ANC type, intention to use family planning was no longer a statistically significant variable.

Women who participated in group ANC had higher odds of using a modern family planning method than those in individual care at one year postpartum, controlling for intention, age, gravida, religion, and highest level of education (AOR = 8.063, CI:2.887,22.524). Immediate post-partum intention to use family planning did not have a significant effect on actual use of modern family planning.

### Exclusive breastfeeding

A greater portion of women who participated in group ANC reported exclusively breastfeeding for more than 6 months than those in individual care (75.5% vs. 50%, X^2^ = 10.94, *p* < .001). Women enrolled in group ANC had nearly three-fold odds of exclusive breastfeeding for more than 6 months compared with women in individual care, controlling for covariates (AOR = 2.84, 95% CI: 1.298, 6.216) (Table 4).

**Table 4:** Adjusted odds ratios for use of family planning methods and breastfeeding

	Family Planning: Modern or non-modern methods		Family Planning: Modern method only		Exclusive breastfeeding: longer than 6 months postpartum	
	Adjusted Odds Ratio	95% Confidence Interval	Adjusted Odds Ratio	95% Confidence Interval	Adjusted Odds Ratio	95% Confidence Interval
Group Care	**6.690**	2.724, 16.420	**8.063**	2.887, 22.524	**3.796**	(1.558, 9.247)
Intentions to Use FP	1.549	0.676, 3.549	1.085	0.444, 2.655	.953	(.400, 2.272)
Age	1.105	0.923, 1.117	.993	0.895, 1.101	.956	(.864, 1.058)
Number of Pregnancies	1.003	0.731, 1.376	1.192	0.851, 1.670	1.028	(.740, 1.427)
Being Muslim (as compared to Christians)	1.222	0.471, 3.168	1.508	0.537, 4.235	.488	(.186, 1.281)
Highest Level of Education (compared to primary education):						
Junior High School	2.656	0.854, 8.267	2.726	0.798, 9.319	1.029	(.323, 3.281)
Senior High School	3.307	0.841, 12.997	4.466	1.062, 18.778	.926	(.206, 4.162)
Tertiary Education	3.452	0.838, 14.218	2.665	0.595, 11.926	.273	(.064, 1.161)

AOR with *p* < .05 in **bold**

## Discussion

While little research exists on group ANC in low- and middle-income countries, several studies have shown the feasibility of delivering group ANC in these settings [26, 27]. Challenges to implementing group ANC in a low resource setting (Haiti) have been described and include space, literacy, language, and cultural appropriateness of content [31]. While this is not the focus of this article, the description of the process of adaptation and implementation are described elsewhere [26, 28].

The impact of group care on family planning in a cohort of 3637 U.S. women found higher odds of family planning use among women participating in group care verses individual care at 3, 6, 9 and 12-months postpartum [32]. Results from our study indicate group ANC has the potential to be an effective model for improving the uptake and continuation of post-partum family planning up to one-year in low resource countries.

Exclusive breastfeeding is recommended by WHO for all newborns up to 6 months of age [33]. The merits of lactational amenorrhea method (LAM) for pregnancy spacing is often promoted in sub-Saharan Africa. A seminal study on the continued contraceptive efficacy of lactational amenorrhea found the 12-month pregnancy rate to be 6% among sexually active, amenorrheic, breast-feeding women not using another form of contraception [34]. Women in our study enrolled in group care had greater odds of exclusive breastfeeding longer than 6 months postpartum. A systematic review and meta-analysis of the effects of CenteringPregnancy (a model of group antenatal care) on breastfeeding in the U.S., found CenteringPregnancy was an effective intervention to increase breastfeeding initiation [19].

There are several limitations to this research. The sample size is small and limited to a single-center. We were only able to locate 68.3% of women enrolled in our study at one-year postpartum. Because exclusive breastfeeding was self-reported from the previous six months, there is the potential of recall bias by the participants. Another limitation to this analysis is a lack of baseline assessment of family planning desire. While women in the intervention and control groups were similar in education, parity, age and religion, without a baseline assessment we cannot be guaranteed that women in group ANC did not begin care during their pregnancy with a higher intention to use contraception postpartum. The wide confidence interval of these estimates reveals uncertainty in the actual magnitude of the effect of these exposures as well as reflect our small sample size.

Despite these limitations, our study employed a rigorous observational research design. Our findings provide evidence on the potential of group ANC as a strategy to increase uptake of postpartum family planning and deserve further attention in future studies with larger sample sizes.

Postpartum family planning has numerous benefits for the health and well-being of mothers and infants. Family planning can delay first pregnancy, lower the risk of unsafe abortion, and reduce the consequences for women of short pregnancy intervals and high parity [35]. Analytic modeling of maternal deaths using data from 172 countries found contraceptive use averted 272,040 maternal deaths, reducing maternal deaths by 44% worldwide with data extracted from 2010 databases [35]. Contraceptive use also has the potential to improve perinatal outcomes by decreasing risks to neonates associated with short pregnancy intervals, such as prematurity and low birthweight [36].

## Conclusion

This is the first study to examine the family planning intent and use at one-year postpartum in women attending group ANC in a low-resource setting. Family planning is a key priority for the government of Ghana as described in their national development policy framework [37]. Enhancing family planning education through ANC enables women to make informed choices about their future sexual and reproductive health. Contraceptive use and child spacing improves maternal and newborn health. Little research exists on integrating family planning services into maternity care. Improving the quality of messaging during ANC has the potential to improve uptake and long-term use of family planning methods postpartum.

## Abbreviations

ANC: Antenatal care
DHS: Demographic & health survey
LAM: Lactational amenorrhea method
RA: Research assistant
